# Development and multi-platform validation of a pan-genomic-driven PMA-qPCR method for the precise quantification of viable *Bifidobacterium animalis subsp. lactis* in complex probiotic formulations

**DOI:** 10.3389/fmicb.2026.1856445

**Published:** 2026-06-24

**Authors:** Lizheng Guo, Jianguo Zhu, Chengyu Song, Mingming Zhu, Yingxin Jiao, Zhiquan Song, Yuanyuan Ge, Xiaoying Jiang, Su Yao

**Affiliations:** 1 China National Research Institute of Food and Fermentation Industries Co., LTD., China Center of Industrial Culture Collection, Beijing, China; 2 Wecare Probiotics Co., Ltd., Suzhou, China

**Keywords:** *Bifidobacterium animalis subsp. lactis*, pan-genomic analysis, PMA-qPCR, validation, viable cell quantification

## Abstract

Accurate quantification of viable *Bifidobacterium animalis* subsp. *lactis* is essential for probiotic quality and functionality, yet remains challenging in complex multi-strain products and diverse matrices. Here, we developed a pan-genomic-driven propidium monoazide–quantitative PCR (PMA-qPCR) assay, enabling robust and precise enumeration of viable *B. lactis* in both single-strain and complex formulations. Using a pan-genomic approach combined with two-tiered in silico and experimental validation, we identified a novel subspecies-specific target, the pyridoxal phosphate homeostasis protein, which allowed exclusive detection without cross-reactivity among 36 non-target strains, including closely related taxa. The optimized PMA protocol demonstrated high strain-independent universality, effectively excluding signals from non-viable cells while maintaining sensitivity (LOD 10^3^ CFU/mL) and strong linearity (R^2^ > 0.99, 10^3^–10^8^ CFU/mL) across diverse sample matrices and excipients. Importantly, the method enabled accurate quantification of *B. lactis* in complex multi-strain probiotic products, and in long-stored samples, PMA-qPCR detected higher viable counts than traditional plate counting; this was corroborated by flow cytometry and single-cell D₂O-labeled Raman spectroscopy, revealing physiologically intact cells beyond culturability. Collectively, these findings establish the pan-genomic-driven PMA-qPCR as a culture-independent, high-resolution tool for reliable quality control and label verification of *B. lactis*, supporting standardized testing, regulatory compliance, and multidimensional viability assessment in increasingly complex commercial probiotic products.

## 1 Introduction

Probiotics are defined by the World Health Organization (WHO) as “live microorganisms that, when administered in adequate amounts, confer a health benefit on the host” (Hill et al., 2014; FAO/WHO, 2002). Among commercially relevant probiotic taxa, *Bifidobacterium animalis* subsp. *lactis* is one of the most extensively documented and widely applied species, owing to its demonstrated roles in modulating gut microbiota, enhancing immune responses, and alleviating gastrointestinal disorders (Uusitupa et al., 2020; Claudio et al., 2017; Saxelin et al., 2005; Plaza-Diaz et al., 2019). Consequently, *B. lactis* is frequently incorporated into functional foods, dietary supplements, and clinical formulations.

Viable probiotic levels are commonly expressed as colony-forming units (CFU). The health benefits of probiotics are strictly dose-dependent, and international regulatory bodies typically require a minimum viable count (often 10^6^–10^7^ CFU/g or mL) throughout the product’s shelf life to ensure efficacy (Tripathi and Giri, 2014). Therefore, accurate quantification of viable cells is paramount for quality assurance, label claim verification, and regulatory compliance in the probiotic industry. Traditionally, the plate count method has been the “gold standard” for enumeration. However, this approach is time-consuming, labor-intensive, and lacks taxonomic specificity, as it cannot easily distinguish target strains from other microbiota in multi-strain products (Postollec et al., 2011; Zawistowska-Rojek et al., 2022; Vinderola et al., 2019). More importantly, plate counting fails to detect cells in the viable but non-culturable (VBNC) state—a physiological condition where bacteria remain metabolically active but do not form colonies on agar, which often leads to a significant underestimation of the true viable population (Pazos-Rojas et al., 2024; Boyte et al., 2023).

To address these limitations, culture-independent molecular approaches, particularly quantitative PCR (qPCR), have been increasingly adopted for rapid and specific detection (Postollec et al., 2011). Nevertheless, conventional qPCR cannot discriminate between DNA originating from viable and dead cells (Zhang et al., 2020). The incorporation of propidium monoazide (PMA) prior to qPCR has been proposed as an effective strategy to overcome this limitation (Nocker et al., 2007; Emerson et al., 2017). PMA selectively penetrates membrane-compromised cells and irreversibly binds to DNA upon photoactivation, thereby preventing amplification from non-viable cells and enabling the preferential quantification of membrane-intact cells (Nocker et al., 2006).

Despite its promise, the application of PMA-qPCR to *B. lactis* in commercial probiotic products remains challenging. The high genomic similarity between *B. lactis* and its close relative, *B. animalis* subsp. *animalis*, severely limits the discriminatory power of commonly used genetic markers. Specifically, these two subspecies exhibit nearly identical sequences in the 16S rRNA gene (often exceeding 99% similarity), and even highly conserved housekeeping genes frequently lack sufficient polymorphism to provide reliable differentiation. Such taxonomic proximity necessitates the discovery of novel genetic signatures beyond traditional markers. Moreover, complex product matrices containing prebiotics, excipients, and high loads of non-target microorganisms can compromise DNA extraction efficiency and PMA performance, underscoring the need for rigorously validated, matrix-resilient methods (Scariot et al., 2018; Dias et al., 2020).

In this study, we established a comprehensive and standardized pan-genomic-driven PMA-qPCR framework for the precise quantification of viable *B. lactis*. A novel, highly specific target gene—pyridoxal phosphate homeostasis protein—was identified through extensive pan-genomic screening and validated across 20 target strains and 36 non-target species following ISO 16140-2:2016 (E) guidelines (ISO 16140-2, 2016). The methodology was rigorously evaluated for its sensitivity, linearity, and accuracy. Moreover, we applied this optimized method to diverse commercial probiotic products and cross-validated the presence of VBNC cells using single-cell heavy water (D₂O)-labeled Raman spectroscopy and flow cytometry. This research provides a sophisticated and reliable tool for the quality control and label verification of *B. lactis* in the increasingly complex probiotic market.

## 2 Materials and methods

### 2.1 Design and validation of specific primers for *Bifidobacterium lactis*

Primers for *B. lactis* were designed using a pan-genomic approach. Genome sequences of *B. animalis*, including closely related *B. animalis* subsp. *animalis* and *B. lactis* strains, were retrieved from the National Center for Biotechnology Information (NCBI) database^1^ following quality control and average nucleotide identity (ANI) analysis (Arahal, 2014). The genomes were re-annotated with Prokka v1.14.6 to identify protein-coding sequences (Seemann, 2014). Subsequently, gene clustering analysis was performed using CD-HIT software (v4.6) with a 50% pairwise identity threshold and a 0.7 length difference cutoff for amino acid sequences (Fu et al., 2012), followed by pan-genome and core-genome analysis. A pan-genome analysis of all *B. animalis* genomes identified 4,517 pan genes and 812 species-level core genes. When the analysis was restricted to *B. lactis* genomes, 3,118 pan genes and 1,068 subspecies-level core genes were identified. Comparative genomic analysis further identified 256 conserved core genes specific to *B. lactis* and differentiated from other subspecies as candidate targets for primer design. The nucleotide specificity of these candidate genes was further confirmed using BLASTN against the NCBI Nucleotide Collection (NT; Altschul et al., 1990). This process revealed the gene encoding the Pyridoxal phosphate homeostasis protein as a species-specific target, which was then used as the basis for primer design. The PCR primer pairs for *B. lactis* were carefully designed using Primer Premier v6.0, following established design guidelines (Singh et al., 1998; Elsalam, 2003). The selected primer set, targeting a 184 bp fragment (Bal-F: 5’-GCGTATGACTTCGACCTGAC-3′; Bal-R: 5’-CGATCTTCTCCGCCAATTCAA-3′), was synthesized by Sangon Biotech (Shanghai, China).

The inclusivity and exclusivity of the primers were evaluated to assess their ability to specifically detect *B. lactis* following the guidelines of ISO 16140 (ISO 16140-2, 2016). A total of 20 different strains of *B. lactis* and 36 strains from the Chinese list of cultures authorized for use in food products were tested (Table 1). The PCR reaction mixture was prepared in a total volume of 50 μL, consisting of 2 μL of DNA template, 2 μL of each primer (10 μmol/L), 25 μL of 2 × PCR Taqmix, and 19 μL of double-distilled water (ddH₂O). The thermal cycling conditions were as follows: an initial denaturation step at 95 °C for 5 min, followed by 35 cycles of denaturation at 95 °C for 30 s, annealing at 60 °C for 34 s, and extension at 72 °C for 25 s, with a final extension at 72 °C for 10 min. The amplification products were visualized using electrophoresis on a 1% agarose gel and analyzed with the Gel Doc EZ System (Bio-Rad, California, USA).

**Table 1:** Bacterial strains used for inclusivity and exclusivity study.

Genus	Strains	Tests
*Bifidobacterium*	*B. lactis* CICC 24210^T^, M8, Bi07, BAL06, BI-04, BB-12, HN019, A-6, UABLa-12, and B420-400B, CICC 10140R, CICC 10190R, CICC 10142R, CICC 6167, CICC 10039, CICC 21712, CICC 24672, CICC 21709, CICC 21710, and CICC 21714	Inclusivity
*Lacticaseibacillus*	*L. paracasei* CICC6263^T^, *L. casei* CICC 6117^T^, *L. rhamnosus* CICC 6224^T^	Exclusivity
*Bifidobacterium*	*B. animalis* subsp. *animalis* CICC 6250^T^, *B. adolescentis* CICC 6070^T^, *B. breve* CICC 6079^T^, *B. longum* subsp. *longum* CICC 6186^T^, *B. longum* subsp. *infantis* CICC 6069^T^, *B. bifidum* CICC 6071^T^	
*Lactobacillus*	*L. acidophilus* CICC 6081^T^, *L. crispatus* JCM 1185^T^, *L. delbrueckii* subsp. *bulgaricus* CICC 6103^T^, *L. delbrueckii* subsp. *lactis* CGMCC 1.2625^T^, *L. gasseri* CICC 24878^T^, *L. helveticus* CICC 24208^T^, *L. johnsonii* CICC 6252^T^, *L. kefiranofaciens* subsp. *kefiranofaciens* CGMCC 1.3402^T^	
*Limosilactobacillus*	*L. fermentum* CICC 24209^T^, *L. reuteri* CICC 6132^T^	
*Lactiplantibacillus*	*L. plantarum* CICC 6240^T^	
*Ligilactobacillus*	*L. salivarius* CGMCC 1.1881^T^	
*Latilactobacillus*	*L. curvatus* JCM 1096^T^, *L. sakei* CICC 6245^T^	
*Streptococcus*	*S. salivarius* subsp. *thermophilus* CICC 6222^T^	
*Lactococcus*	*L. lactis* subsp. *lactis* CICC 6246^T^, *L. cremoris* CICC 24337^T^	
*Propionibacterium*	*P. freudenreichii* subsp. *shermanii* CGMCC 1.2231^T^, *P. acidipropionici* CICC 24923^T^	
*Leuconostoc*	*L.* subsp. *mesenteroides* CICC 25070^T^, *L. mesenteroides* subsp. *cremoris* CICC 22181	
*Pediococcus*	*P. acidilactici* CGMCC 1.2696^T^, *P. pentosaceus* CGMCC 1.2695^T^	
*Weizmannia*	*W. coagulans* CGMCC 1.2009^T^	
*Staphylococcus*	*S. vitulinus* CICC 10850, *S. xylosus* JCM 2418^T^, *S. carnosus* ACCC 01657	

### 2.2 Validation of PMA protocols for differentiating viable and dead *Bifidobacterium lactis*

To ensure accurate discrimination between viable and dead cells, appropriate PMA treatment conditions are required. Based on previous studies demonstrating effective discrimination between viable and dead probiotic cells, a widely used PMA treatment protocol consisting of 50 μM PMA, 5 min dark incubation, and 15 min light exposure was adopted in this study (Desfossés-Foucault et al., 2012; Villarreal et al., 2013; Guo et al., 2024a; Guo et al., 2024b). To further evaluate the applicability of this condition across different *B. lactis* strains, 20 strains (Table 1) were selected for subsequent analysis. The bacteria were firstly cultured on de Man, Rogosa and Sharpe (MRS) agar (Land Bridge, Beijing, China) at 37 °C for 48 h, then re-inoculated onto fresh plates and incubated for an additional 48 h to ensure they were in an active growth phase. The bacterial suspensions were prepared using 0.85% NaCl solution, with the optical density adjusted to 0.3–0.5 at 620 nm, equating to approximately 10^8^ CFU/mL, as verified by plate counting. To generate dead bacterial samples, the bacteria were subjected to heat treatment at 90 °C for 10 min. The treated cells were subsequently cultured on MRS agar plates, and no colony growth was observed after incubation, confirming successful inactivation of the cells. Both live and dead bacterial suspensions, each with a concentration of 10^8^ CFU/mL, were then divided into two groups: one treated with PMA and one without. A 20 mmol/L stock solution of PMA (from BIORIGIN, China) was added to the bacterial suspensions to achieve a final concentration of 50 μM. After 5 min of dark incubation to allow the PMA to penetrate the dead cells, the samples were exposed to a 60 W LED light source (Biotium, USA) for 15 min. Following light exposure, the suspensions were centrifuged at 12,000 rpm for 15 min, and the resulting bacterial pellets were used for DNA extraction.

DNA extraction was carried out using the bead-beating method, which has been shown to be effective (Fujimoto et al., 2004; Guo et al., 2024a; Guo et al., 2024b). For this, 2.0 mL sample tubes were sterilized and loaded with 0.25 g of 0.1 mm Zirconia/Silica beads. A 200 μL bacterial suspension in ddH₂O was then added to each tube. Cell disruption was achieved by placing the tubes in a BEAD RUPTOR 12 (OMNI International, USA) and running the device at 6.0 m/s for 12 s. After disruption, the samples were centrifuged at 12,000 rpm for 15 min to separate the supernatant containing the DNA. A 50 μL aliquot of the supernatant was transferred into 1.5 mL sterile tubes for subsequent qPCR analysis (Guo et al., 2024a; Guo et al., 2024b).

### 2.3 Construction of standard curves

To enable viable cell quantification using PMA-qPCR, a standard curve correlating viable cell counts with qPCR quantification cycle (Cq) values was established, as described previously (Lv et al., 2016; Tavernier and Coenye, 2015; Rey et al., 2021). Fresh cultures of *B. lactis* CICC 24210 T were prepared and adjusted to approximately 10^8^ CFU/mL, with concentrations verified by plate counting. The bacterial suspension was subjected to PMA treatment to eliminate signals from non-viable cells, followed by DNA extraction as outlined above. Subsequently, a series of 10-fold dilutions of the extracted DNA was prepared and used for qPCR amplification to generate the corresponding C_q_ values. The total qPCR volume was 20 μL per reaction, including 10.0 μL of 2 × SYBR Green premix (TaKaRa, Japan), 0.4 μL each of 10 μM forward and reverse primers, 0.08 μL of ROX reference dye, 2 μL of bacteria genomic DNA, and 7.12 μL ddH_2_O. The thermal cycle program was as follows: 95 °C for 30 s, followed by 40 cycles of 95 °C for 5 s, 60 °C for 34 s. The qPCR reactions were carried out in an ABI 7500 Fast real-time PCR system. Triplicates were performed for both target DNA and sterile water (negative control). Subsequently, the standard curve between C_q_ values and viable cell numbers were constructed.

### 2.4 Comprehensive validation of PMA-qPCR for accurate viable quantification of *Bifidobacterium lactis*

To establish a reliable and accurate PMA-qPCR method for the viable quantification of *B. lactis*, a comprehensive validation study was conducted, addressing four key aspects: strain specificity and consistency in pure cultures, quantitative performance across a range of *B. lactis* concentration, applicability to probiotic raw materials and food ingredients, and performance in complex commercial probiotic products. Following ISO 16140-3 guidelines, an acceptable deviation (△Log₁₀) of ±0.5 between PMA-qPCR results and theoretical values was used as the criterion for accuracy evaluation (ISO 16140-3, 2021).

#### 2.4.1 Evaluation using pure cultures

To assess strain-level applicability and consistency, the PMA-qPCR method was first validated using 20 different *B. lactis* strains (Table 1). These included the type strain CICC 24210^T^ and commercial strains such as M8, Bi-07, BAL06, BI-04, BB-12, HN019, A-6, UABla-12, and B420-400B, along with other strains from the CICC culture collection (e.g., CICC 10140R, CICC 10190R, CICC 21712, etc.). All strains were cultured to a concentration of approximately 10^8^ CFU/mL, then subjected to PMA treatment, DNA extraction, and qPCR analysis. Plate counting was conducted in parallel as a reference method to determine the viable counts.

#### 2.4.2 Quantitative accuracy and detection limits across a range of *Bifidobacterium lactis* concentrations

To evaluate the quantitative accuracy and limit of detection of the PMA-qPCR method under mixed microbial conditions, artificial samples were prepared containing a fixed number (~10^8^ CFU/mL) of heat-inactivated *Lacticaseibacillus paracasei* cells and a gradient of viable *B. lactis* cells (ranging from 10^8^ to below 10^3^ CFU/mL). This design simulated potential matrix interference from non-target or non-viable bacteria. Culture-based plate counting was used to determine the theoretical concentrations of viable *B. lactis*, and the PMA-qPCR measurements were compared to these values.

#### 2.4.3 Application to probiotics as food ingredient

The practical applicability of the PMA-qPCR method for viable quantification in food matrices was assessed using five commercial probiotic formulations in powder form. These samples included either pure *B. lactis* strains (e.g., Bi07, HN019, v9) or compound formulations containing *B. lactis* alongside other probiotic species, blended with excipients such as maltodextrin. Samples were rehydrated and diluted to approximately 10^8^ CFU/mL prior to analysis.

#### 2.4.4 Application to commercial probiotic products

The commercial probiotic products analyzed in this study exhibited substantial complexity at both the microbial and matrix levels, providing a representative and challenging context for method evaluation.

From the perspective of microbial composition, these products typically consisted of multi-genus and multi-strain consortia. The dominant taxa included species from the genera *Lactobacillus*, *Bifidobacterium*, and *Streptococcus*, often accompanied by other lactic acid bacteria. Notably, several formulations included multiple strains of *B. lactis* (e.g., HN019, BI-04). As these strains belong to the same subspecies, they are collectively targeted by the assay and do not interfere with subspecies-level quantification.

From the perspective of matrix composition, the excipient systems were highly heterogeneous and comprised diverse functional ingredients. These included prebiotic carbohydrates such as resistant dextrin, polydextrose, fructo-oligosaccharides, galacto-oligosaccharides, and isomalto-oligosaccharides; sugar alcohols such as erythritol, maltitol, and lactitol; protein-rich and dairy-derived components such as whole milk powder and whey protein; as well as plant-derived ingredients including fruit powders and botanical extracts. In addition, various functional additives (e.g., inulin, stachyose, chitosan oligosaccharides) and mineral fortificants (e.g., calcium and zinc compounds) were present in certain formulations.

Such complexity introduces multiple analytical challenges for nucleic acid-based detection methods. Specifically, the presence of high-background DNA from non-target strains may interfere with assay specificity, while diverse matrix components may act as PCR inhibitors. Moreover, the coexistence of multiple closely related strains further increases the difficulty of achieving accurate and selective quantification. Therefore, these products constitute a stringent test system for evaluating the specificity, accuracy, and robustness of the PMA-qPCR method for viable *B. lactis* detection in real-world applications.

### 2.5 Multi-method detection of VBNC probiotics in long-term stored sample

For probiotic products subjected to long-term storage, the constituent bacteria may enter a VBNC state. In this study, a probiotic sample, which had been stored at −20 °C for 3 years, was analyzed to investigate this phenomenon. Multiple techniques, including traditional plate counting, the established PMA-qPCR method, flow cytometry (FCM), and D₂O-labeled single cell Raman spectroscopy (D₂O-SCRS), were employed to investigate the presence of VBNC cells.

The 25 g sample was added to 225 mL of a 0.85% NaCl solution, and the bacterial concentration was diluted to approximately 10^8^ CFU/mL. The viable cell count was then determined using the established PMA-qPCR method. The sample was further diluted to appropriate gradients, and the cultivable cell count was assessed using MRS agar plates.

For flow cytometry analysis, SYTO9 and propidium iodide (PI) were used to distinguish live and dead cells. A 980 μL aliquot of bacterial suspension at 10^6^ CFU/mL was mixed with 10 μL of a 0.1 mM SYTO9 working solution and 10 μL of a 0.2 mM PI working solution. The mixture was vortexed for 30 s, incubated in the dark for 15 min, and analyzed using a flow cytometer (BD Accuri™ C6, BD, USA).

For D₂O-labeled Raman spectroscopy, 10^8^ CFU/mL of bacteria were incubated in 100% D₂O culture medium at 37 °C for 1 h, with an additional group without D₂O as a control. After incubation, the bacterial suspension was washed three times with sterile ultrapure water. The suspension was resuspended, and 5 μL was dropped onto a Raman microscope slide and air-dried. Raman spectra were then collected using a Raman spectrometer (PRECI SCS-R300, Hooke, China).

### 2.6 Statistical analysis

The T-test, performed using Excel (Microsoft Office, 2021), was used to evaluate the significance of PMA treatment on viable cell counts. The Wilcoxon rank-sum test was employed to compare results obtained from the plate counting method with those from PMA-qPCR, flow cytometry, and D₂O-labeled Raman spectroscopy. A *p* value of < 0.05 was considered statistically significant.

## 3 Results

### 3.1 Specificity and inclusivity of the pan-genomic-driven primers

**Figure 1:**
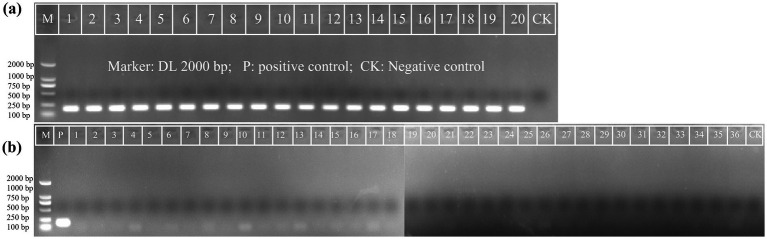
PCR amplification results demonstrating the specificity of the newly designed primer on an agarose gel. **(a)** Amplification of DNA from 20 *B. lactis* target strains (Table 1), confirming inclusivity. **(b)** Testing of 36 non-target strains (Table 1), all of which showed no amplification, verifying exclusivity.

The specificity of the newly designed primer set was evaluated through comprehensive inclusivity and exclusivity tests using DNA templates from 20 *B. lactis* strains and 36 non-target strains. Consistent with the in-silico predictions, positive amplification was observed exclusively in the 20 target strains, each yielding a single 184 bp amplicon. In contrast, no amplification was detected in any of the 36 non-target strains, including closely related subspecies (Figure 1). These findings confirm the exclusive specificity of the primers for *B. lactis*, enabling precise differentiation at the subspecies level. Such high resolution is particularly advantageous for the accurate quantification of *B. lactis* within complex microbial consortia and probiotic formulations.

### 3.2 Efficiency and strain-level robustness of the PMA treatment

**Figure 2:**
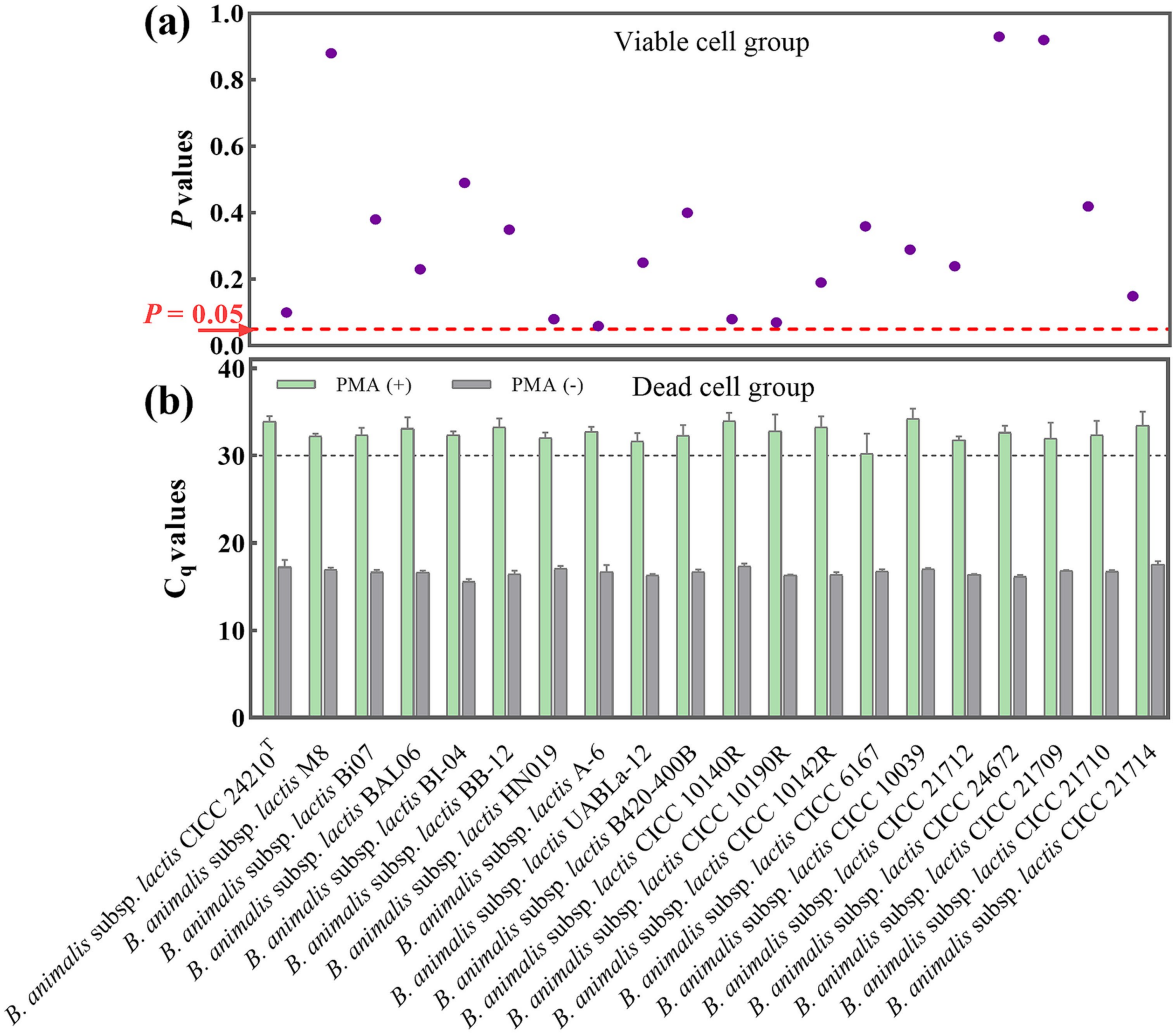
Performance of the optimized PMA protocol across 20 *B. lactis* strains. **(a)** Comparison of Cq values between PMA-treated (+) and untreated (−) viable cells, showing high compatibility with DNA amplification across genetically diverse strains. **(b)** Efficiency of PMA treatment in neutralizing DNA from heat-inactivated (dead) cells, confirming the complete elimination of non-viable cell signals. Error bars represent the standard deviation of triplicate measurements.

To effectively discriminate viable *B. lactis* from membrane-compromised cells, PMA treatment conditions were evaluated and validated across 20 genetically diverse strains. Based on preliminary optimizations, a final PMA concentration of 50 μM, followed by 5 min of dark incubation and 15 min of light exposure, was employed. To ensure that these parameters did not interfere with the detection of viable populations, the 20 *B. lactis* strains were analyzed with and without PMA treatment. Statistical analysis of the resulting Cq values showed no significant differences (*p* > 0.05, with *p*-values ranging from 0.06 to 0.93) between the treated and untreated viable cells (Figure 2a). This confirms that the selected PMA concentration and light-exposure duration do not inhibit the qPCR amplification of DNA from viable cells.

The efficacy of PMA in suppressing DNA signals from dead cells was further rigorously tested. All 20 strains were heat-inactivated (10^8^ CFU/mL) and subjected to the same PMA-qPCR protocol. As illustrated in Figure 2b, the Cq values for PMA-treated dead cells consistently exceeded 30 cycles (or showed no amplification) across all strains, indicating that DNA from non-viable cells was effectively neutralized. The inhibition efficiency reached 100%, demonstrating that the optimized protocol provides a high degree of selectivity. These findings confirm that the established PMA treatment effectively prevents the amplification of DNA from dead *B. lactis*, ensuring the reliable quantification of viable cells across a broad range of type and commercial strains.

### 3.3 Construction of the standard curve and evaluation of amplification efficiency

**Figure 3:**
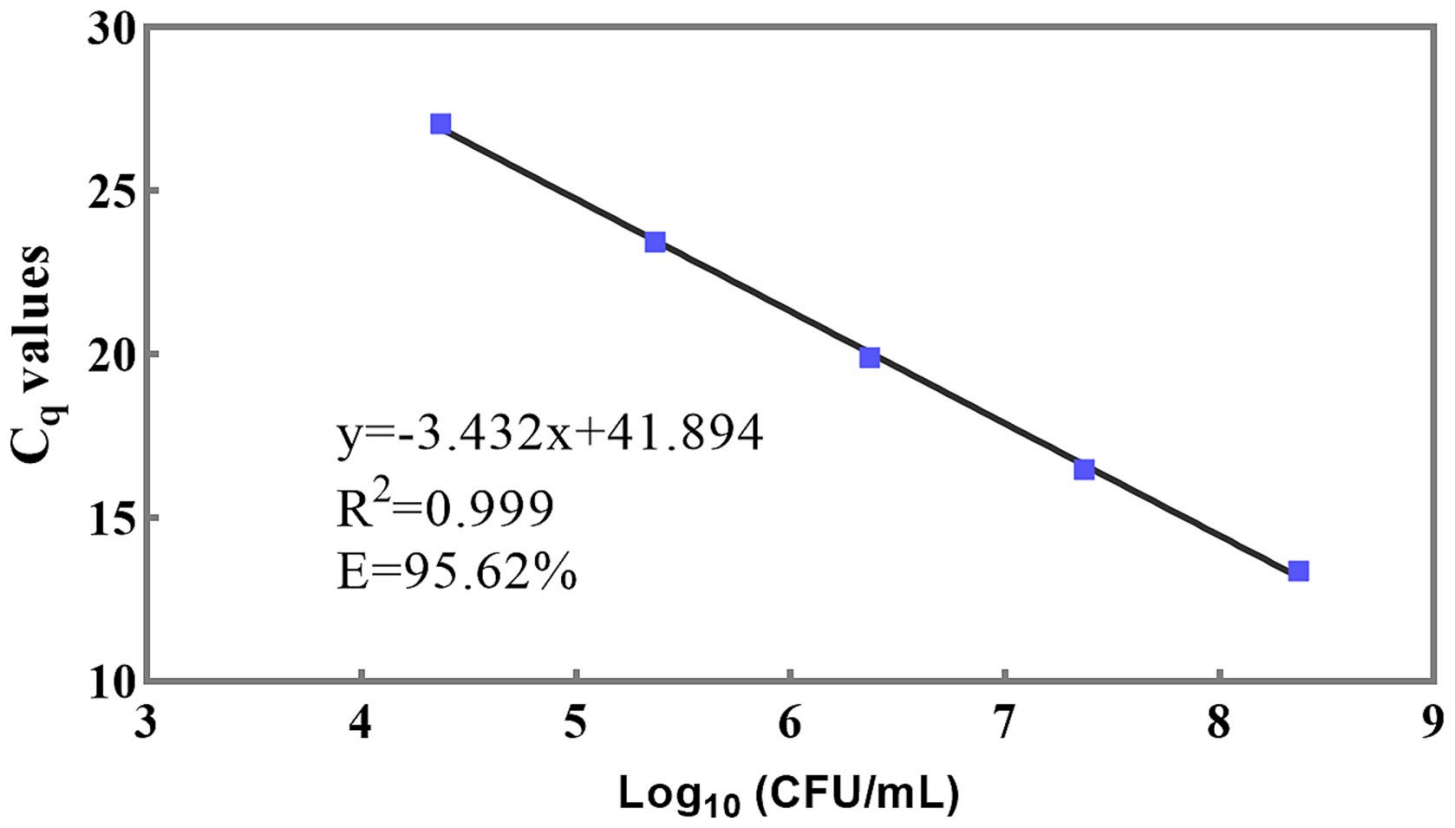
Quantitative sensitivity and standard curve of the *B. lactis*-specific qPCR assay. The standard curve illustrates the linear relationship between the mean Cq values and the log-transformed concentrations of culturable *B. lactis* (Log CFU/mL). Data points were derived from 10-fold serial dilutions of target DNA, with each point representing the mean of triplicate measurements.

In qPCR analysis, the quantification of target DNA is fundamentally based on the Cq value. To facilitate the enumeration of viable cells, a robust correlation between Cq values and viable *B. lactis* concentrations was established (Figure 3). The linear regression analysis, correlating Cq values with the logarithm of viable cell counts (log CFU/mL), yielded a slope of −3.432 and a high coefficient of determination (R^2^ = 0.999). Based on the formula *E* = 10 ^(−1/slope)^ – 1, the PCR amplification efficiency (E) was determined to be 95.62% (Svec et al., 2015; Rogers-Broadway and Karteris, 2015). This value falls well within the internationally accepted range of 90 to 110% (Ruijter et al., 2009), underscoring the high sensitivity and optimized performance of the newly designed primers. Consequently, this established standard curve provides a reliable framework for converting Cq values into CFU-equivalent viable cell counts, ensuring high precision for *B. lactis* quantification in subsequent sample analyses.

### 3.4 Methodological robustness across diverse sample types

#### 3.4.1 Validation in pure cultures and strain-level applicability

**Figure 4:**
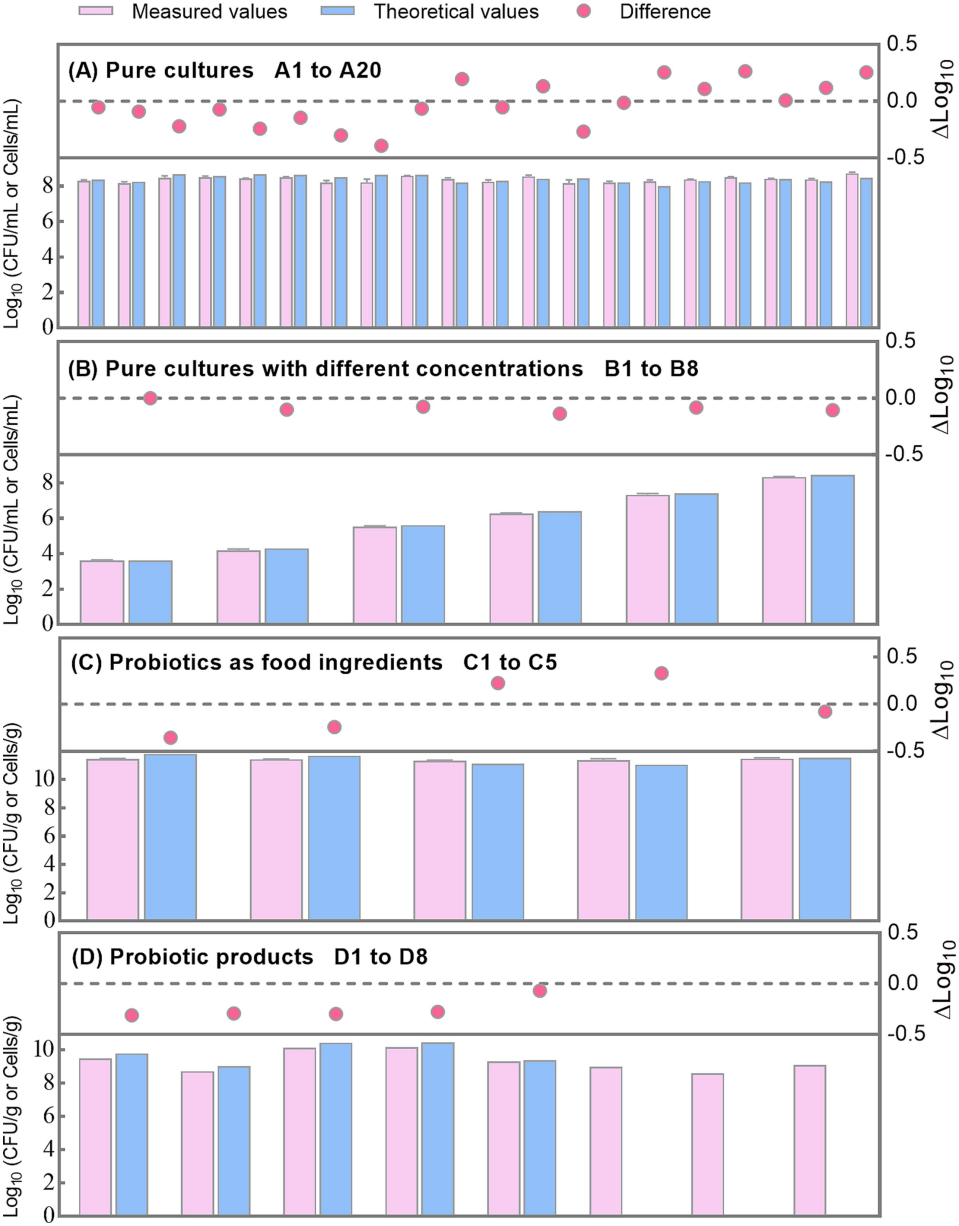
Accuracy and applicability of the PMA-qPCR method across diverse sample matrices. The graphs illustrate the agreement between PMA-qPCR quantified results and theoretical values (determined by plate counting or manufacturer specifications). **(A)** A1–A20: 20 different strains of *B. lactis* in pure culture; **(B)** B1–B6: serial bacterial suspensions ranging from 10^3^ to 10^8^ CFU/mL; **(C)** C1–C5: five probiotic raw materials used as food ingredients; **(D)** D1–D8: eight commercial multi-strain probiotic products. The dashed lines indicate the ±0.5 Log_10_ acceptance limits in accordance with ISO 16140-3, demonstrating the method’s robust accuracy and matrix tolerance.

Initially, pure cultures were analyzed to establish a baseline free of matrix interference. The deviations between the PMA-qPCR measurements and theoretical values (determined by plate counting) ranged from −0.394 to 0.263 Log₁₀ (Figure 4A), confirming high quantification accuracy. Notably, these results validated that the standard curve derived from the reference strain *B. lactis* CICC 24210^T^ is universally applicable to other commercial strains. This finding has significant practical implications, as it eliminates the necessity for constructing strain-specific standard curves for the quantification of viable *B. lactis* in diverse commercial formulations.

#### 3.4.2 Linear dynamic range and sensitivity

The quantitative range of the method was evaluated using bacterial suspensions across a gradient of 10^3^ to10^8^ CFU/mL. Within this dynamic range, the △Log₁₀ values varied from −0.1369 to 0.0004 (Figure 4B), remaining well within the predefined acceptance threshold. Linear regression analysis between the measured and theoretical values yielded the equation *y* = 0.98*x* + 0.013 (R^2^ = 0.998), indicating an excellent correlation. The lower limit of quantification (LOQ) was determined to be 10^3^ CFU/mL, establishing a validated dynamic range of 10^3^ to 10^8^ CFU/mL. For samples exceeding this range, appropriate dilution is recommended prior to analysis.

#### 3.4.3 Performance in probiotic raw materials and food ingredients

Probiotic raw materials, typically consisting of high-potency bacterial powders blended with simple excipients (e.g., maltodextrin or resistant dextrin), were tested to assess matrix resilience. As illustrated in Figure 4C, the △Log₁₀ values ranged from **−**0.356 to 0.328, indicating high measurement accuracy and significant tolerance to common carrier matrices. These results support the method’s suitability for quality control during the screening and industrial production of probiotic ingredients.

#### 3.4.4 Performance in complex commercial multi-strain probiotic products

The robustness of the PMA-qPCR method was further evaluated using eight commercially available multi-strain probiotic products with complex compositions. Among the eight products tested, five declared the presence of *B. lactis* on their labels. For these products, the △Log₁₀ values ranged from −0.314 to −0.071 (Figure 4D), demonstrating good agreement with labeled values and confirming the method’s accuracy under complex multi-strain conditions. The presence of numerous co-existing probiotic taxa did not interfere with target quantification, highlighting the strong specificity of the pan-genome-based primer design at the subspecies level.

Furthermore, the method exhibited high tolerance to a wide range of excipient matrices, including prebiotic carbohydrates (e.g., FOS, raffinose), sugar alcohols (e.g., erythritol), and plant-derived powders (e.g., cranberry, peach, and hawthorn). No significant matrix-associated interference was observed.

Notably, viable *B. lactis* was detected in three products lacking explicit declaration of the added amount of this subspecies. This highlights the capability of the PMA-qPCR method to estimate viable cell levels in products with incomplete labeling, supporting its application as a reliable approach for probiotic content assessment under such conditions.

### 3.5 Comparative analysis and correlation of PMA-qPCR with standard plate counting

**Figure 5:**
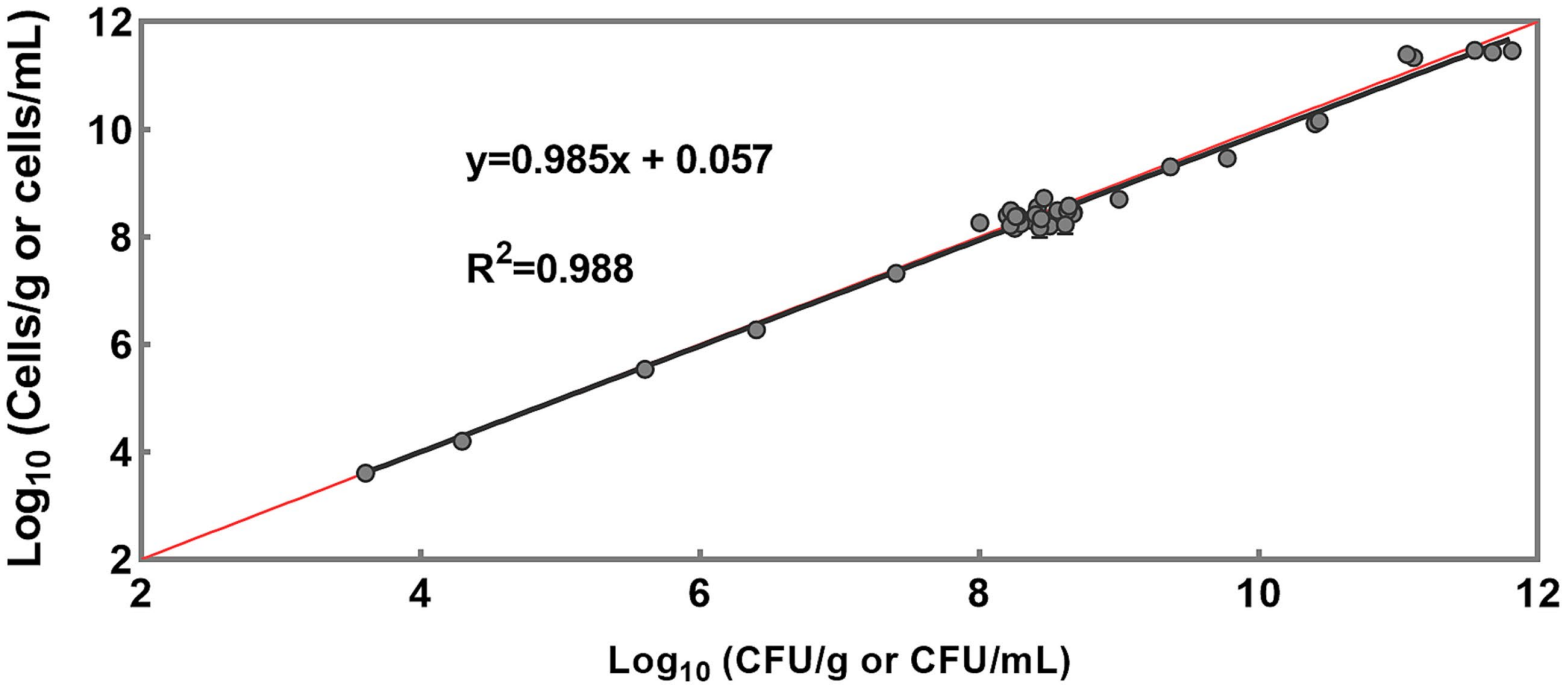
Correlation and agreement between PMA-qPCR counts and theoretical values across various sample types. The red line represents the identity line (1:1 relationship), while the black line indicates the linear regression. The proximity of the regression line to the identity line demonstrates the high accuracy and equivalence of the PMA-qPCR method compared to traditional plate counting.

As illustrated in Figure 5, the ratio of measured to theoretical values closely approximated 1:1, with the linear regression curve nearly overlapping the identity line (y = x). The coefficient of determination (R^2^ = 0.988) indicates a strong linear correlation and a high degree of equivalence between the two methods.

These findings demonstrate that the established PMA-qPCR method yields quantification results highly consistent with those of traditional plate counting, supporting its potential as a reliable alternative for viable probiotic enumeration. Given the inherent limitations of plate counting—such as prolonged turnaround times, high labor intensity, and the inability to distinguish specific target strains within mixed microbial communities—PMA-qPCR offers a more rapid, specific, and high-throughput diagnostic tool. The high equivalence observed in this study provides a robust foundation for the future standardization of PMA-qPCR in food microbiological quality assessments. Furthermore, this method could significantly enhance regulatory oversight and product label verification, particularly for complex multi-strain probiotic formulations that are increasingly prevalent in the commercial market.

### 3.6 Evaluation of probiotic viability and VBNC states during extended storage via multi-platform analysis

**Figure 6:**
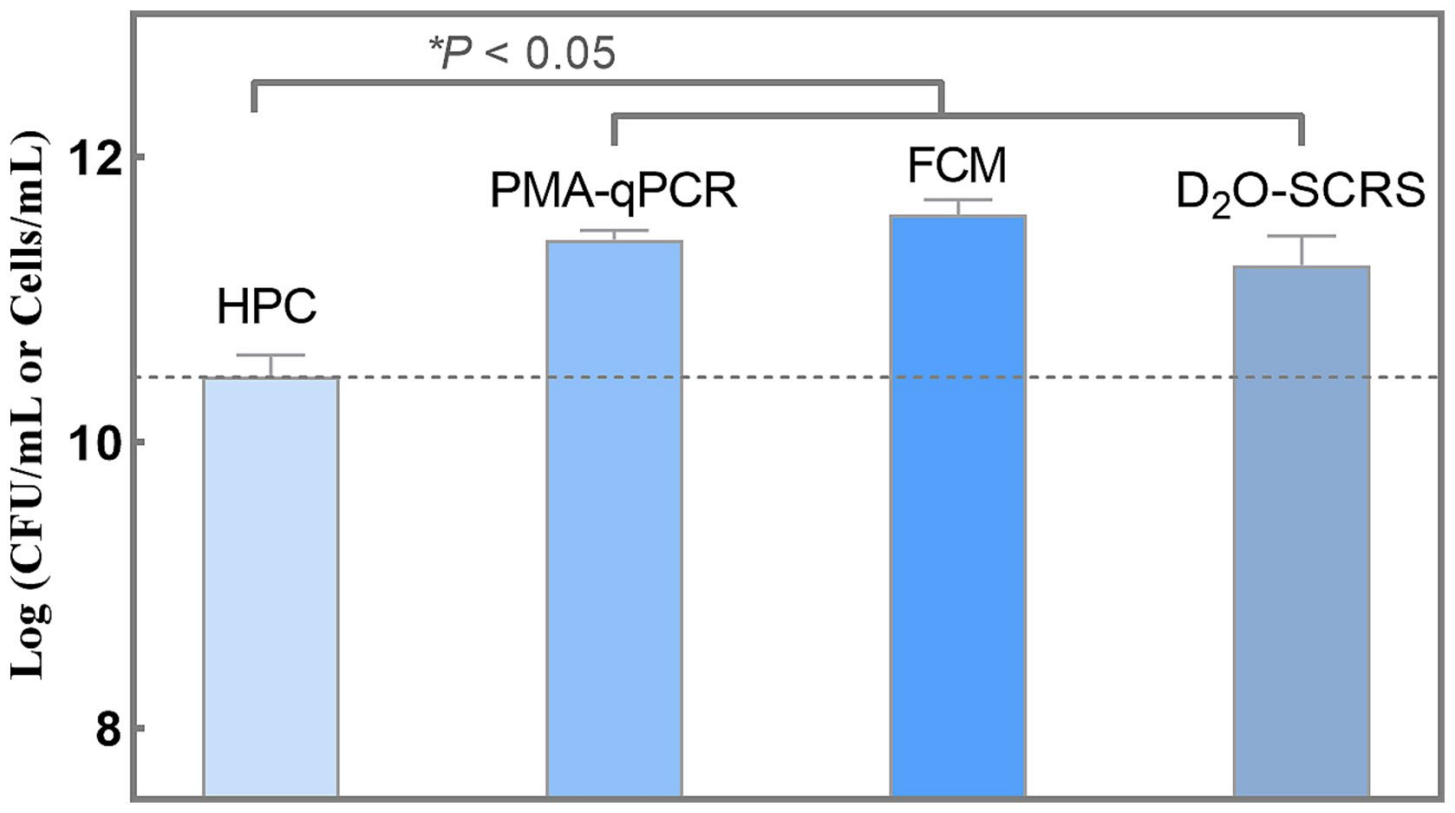
Multi-platform comparative quantification of viable *B. lactis* in a three-year stored probiotic product. Viable counts were assessed via plate counting, PMA-qPCR, flow cytometry, and D_2_O-labeled Raman spectroscopy. The significant discrepancy between culture-dependent and culture-independent methods (*p* < 0.05) highlights the presence of a substantial VBNC population. Error bars represent the standard error of the mean.

A commercial probiotic product stored at −20 °C for 3 years was analyzed using four independent analytical platforms, including standard plate counting, PMA-qPCR, FCM, and D₂O-SCRS methods. The viable counts obtained were 10.46 ± 0.16 Log₁₀ (plate count), 11.42 ± 0.06 Log₁₀ (PMA-qPCR), 11.59 ± 0.11 Log₁₀ (FCM), and 11.24 ± 0.21 Log₁₀ (D₂O-SCRS). Compared to the plate count, the deviations (∆Log₁₀) for the alternative methods were 0.96, 1.13, and 0.78, respectively. Notably, all three culture-independent methods yielded significantly higher viable counts than plate counting (*p* < 0.05), demonstrating high consistency among the alternative platforms (Figure 6).

These findings suggest that during long-term storage, a substantial portion of the *B. lactis* population enters a VBNC state—characterized by compromised culturability but preserved metabolic activity and membrane integrity. Consequently, relying solely on traditional plate counting for aged probiotic products may lead to a significant underestimation of the actual viable population, potentially misrepresenting the product’s functional efficacy.

In contrast, PMA-qPCR, which targets cells with intact membranes regardless of their ability to form colonies, provides a more accurate assessment of the true viable counts in stressed or aged products. This underscores the necessity of integrating advanced molecular or single-cell techniques into routine probiotic quality control frameworks. Furthermore, our results advocate for a re-evaluation of current industry standards by regulatory stakeholders, highlighting the need to adopt detection technologies that more accurately reflect the complex physiological states of probiotics in commercial formulations.

## 4 Discussion

### 4.1 Pan-genomic target selection enables subspecies-level specificity

Accurate quantification of *B. lactis* in complex probiotic formulations remains challenging due to the exceptionally high genomic similarity within the *B. animalis* taxon. Comparative genomic analyses have demonstrated extensive genome conservation among subspecies, rendering commonly used molecular markers such as the 16S rRNA gene and housekeeping genes fundamentally insufficient for reliable discrimination (Briczinski et al., 2009; Janda and Abbott, 2007; Ramasamy et al., 2014).

To overcome this intrinsic limitation, this study adopted a pan-genomic strategy that shifts target selection from single loci to genome-wide screening. This approach enables high-resolution identification by systematically identifying subspecies-specific genetic signatures. By integrating large-scale public genome datasets with stringent protein clustering using CD-HIT (Fu et al., 2012), followed by nucleotide-level validation, a novel subspecies-specific target—the pyridoxal phosphate homeostasis protein gene—was identified.

This multi-tiered in silico framework ensures both inclusivity across *B. lactis* strains and exclusivity against closely related taxa and co-existing probiotic species. Such genome-wide target mining strategies have been shown to substantially improve specificity and reduce cross-reactivity in molecular diagnostics (Laing et al., 2010; Maiden et al., 2013).

From an application perspective, this level of specificity directly supports accurate strain tracking, label verification, and quality control in increasingly complex probiotic formulations. Overall, these findings demonstrate that pan-genomic target selection provides a necessary and scalable solution for subspecies-level detection in closely related bacterial taxa.

### 4.2 Universal PMA treatment ensures robust viability discrimination across strains

Effective PMA treatment is critical for viability-based qPCR, as dye penetration and DNA intercalation are influenced by strain-dependent variations in cell envelope structure and physiological state (Nocker et al., 2007; Fittipaldi et al., 2012). In this study, the optimized PMA protocol demonstrated consistent performance across 20 phylogenetically and commercially representative *B. lactis* strains, thereby explicitly accounting for biological variability among industrial isolates.

The absence of significant differences in Cq values between PMA-treated and untreated viable cells indicates that the optimized conditions preserve membrane integrity without inducing cytotoxic effects (Figure 2a). This observation is consistent with previous studies showing that properly optimized PMA treatment can selectively penetrate membrane-compromised cells while excluding intact viable cells, even in Gram-positive bacteria with complex cell wall structures (Nocker and Camper, 2009; Kramer et al., 2009).

In contrast, DNA signals from heat-killed cells were consistently suppressed across all strains (Figure 2b), confirming effective exclusion of non-viable DNA. This strain-independent performance eliminates the need for protocol customization and ensures reproducibility across different strain backgrounds.

Collectively, these results demonstrate that the optimized PMA treatment provides a robust and universally applicable framework for viability discrimination, supporting standardized implementation in routine analytical workflows.

### 4.3 PMA-qPCR achieves methodological equivalence with plate counting

This study demonstrates that the established PMA-qPCR method achieves methodological equivalence with conventional plate counting while offering significant advantages in efficiency and scalability. Validation was conducted in accordance with the ISO 16140-3:2021 framework, providing internationally recognized criteria for alternative microbiological methods. Across all tested matrices, deviations (ΔLog₁₀) remained within the accepted ±0.5 Log₁₀ range (Figure 4), confirming quantitative agreement under defined conditions. This level of consistency supports the reliability of PMA-qPCR as an alternative enumeration approach in complex probiotic matrices. Nevertheless, DNA extraction efficiency may influence quantitative accuracy in complex matrices due to differences in sample composition and cell disruption efficiency. In this study, a standardized bead-beating DNA extraction method previously validated for probiotic bacteria (Guo et al., 2024a; Guo et al., 2024b) was consistently applied across all samples to minimize extraction variability and improve methodological robustness.

A key advantage for industrial application is the universality of the standard curve. The ability to apply a single calibration curve across multiple *B. lactis* strains reduces analytical variability, simplifies workflows, and enhances operational efficiency. This directly addresses common scalability challenges in probiotic quality control laboratories (Davis, 2014; Salvetti et al., 2018).

Furthermore, the validated dynamic range (10^3^–10^8^ CFU/mL) and strong correlation with plate counting (R^2^ = 0.988; Figure 5) demonstrate robust quantitative performance. Importantly, this equivalence is achieved with a substantially reduced turnaround time, supporting applications in rapid testing, in-process monitoring, and product stability evaluation. More importantly, the method enables selective viable quantification of target strains in complex multi-strain probiotic formulations, which may provide practical advantages for industrial quality control and label verification applications.

### 4.4 Culture-independent approaches reveal multidimensional viability in long-term stored products

Analysis of long-term stored probiotic products revealed substantial discrepancies between culture-based and culture-independent viability assessments. PMA-qPCR, FCM, and D₂O-SCRS consistently reported viable counts approximately 1.0 Log₁₀ higher than plate counting (Figure 6), indicating that a significant fraction of cells retained physiological integrity despite reduced culturability. Notably, the magnitude of this difference suggests a systematic rather than random bias, consistent with previous observations in probiotics subjected to environmental stress and prolonged storage (Lahtinen et al., 2006; Davis, 2014).

Evidence from complementary techniques supports this interpretation at multiple physiological levels. PMA-qPCR selectively quantified cells with intact membranes, reflecting structural integrity, while D₂O-SCRS detected C–D (carbon–deuterium) bands, which indicate deuterium incorporation and directly demonstrate residual metabolic activity at the single-cell level (Berry et al., 2015; Li et al., 2011). Flow cytometry further provided population-level physiological profiling based on membrane permeability and dye responsiveness. Together, these approaches capture distinct yet overlapping dimensions of cellular viability, including membrane integrity, metabolic activity, and physiological responsiveness, whereas plate counting primarily reflects the ability of cells to reproduce under defined culture conditions.

This divergence highlights that the loss of culturability does not necessarily coincide with the loss of cellular integrity or metabolic function. Under prolonged storage and environmental stress, probiotic cells may undergo physiological adaptations that preserve essential cellular structures and basal metabolic activity while impairing their capacity to form colonies on agar media. Such decoupling between reproductive capability and other viability indicators provides a plausible explanation for the observed discrepancies and underscores the multidimensional nature of microbial viability.

These results reveal a fundamental limitation of relying exclusively on culturability as a proxy for probiotic viability, particularly for long-shelf-life products. While plate counting remains indispensable for regulatory purposes, it may systematically underestimate physiologically intact cells and thus fail to capture the full biological potential of probiotic formulations (Aureli et al., 2011). In contrast, PMA-qPCR provides a culture-independent yet operationally accessible assessment based on membrane integrity, offering improved sensitivity to subpopulations that remain structurally intact.

Rather than replacing traditional methods, these findings support a complementary testing paradigm in which culture-independent approaches, particularly PMA-qPCR, augment culture-based enumeration. Such integration enables a more comprehensive and realistic evaluation of probiotic viability and stability, supporting improved quality control, more accurate label verification, and better alignment between analytical measurements and functional cell states. Although this study focused on *B. lactis*, the overall strategy combining pan-genomic target selection with PMA-qPCR may also provide methodological references for the selective viable quantification of other probiotic species and complex multi-strain systems.

## 5 Conclusion

This study established and validated a highly specific and broadly applicable PMA-qPCR method for the viable quantification of *B. lactis* in both single- and multi-strain probiotic products. By integrating a pan-genomic target selection strategy with a two-tiered validation framework, the assay achieved exceptional subspecies-level specificity, enabling accurate detection of the target organism in complex microbial communities without interference from co-existing taxa. The optimized PMA treatment demonstrated strong strain-independent performance across 20 diverse isolates and maintained reliable signal discrimination in heterogeneous sample matrices. Method validation confirmed a high level of agreement with plate counting (R^2^ = 0.988) over a wide dynamic range (10^3^–10^8^ CFU/mL), while also revealing its improved sensitivity in detecting viable cells in long-term stored products. The use of a universal standard curve further enhances its practicality for routine application by reducing analytical variability and operational complexity. Overall, this culture-independent approach provides a robust and scalable tool for probiotic viability assessment, offering improved accuracy in complex formulations and supporting more reliable quality control and label verification. Future work should focus on inter-laboratory validation and integration with complementary analytical techniques to advance standardized, multidimensional evaluation frameworks for probiotic products.

## Data Availability

The original contributions presented in the study are included in the article/supplementary material, further inquiries can be directed to the corresponding author.

## Footnotes

1
https://www.ncbi.nlm.nih.gov/genome/
